# DNA Hypomethylation Affects Cancer-Related Biological Functions and Genes Relevant in Neuroblastoma Pathogenesis

**DOI:** 10.1371/journal.pone.0048401

**Published:** 2012-11-07

**Authors:** Gemma Mayol, José I. Martín-Subero, José Ríos, Ana Queiros, Marta Kulis, Mariona Suñol, Manel Esteller, Soledad Gómez, Idoia Garcia, Carmen de Torres, Eva Rodríguez, Patricia Galván, Jaume Mora, Cinzia Lavarino

**Affiliations:** 1 Developmental Tumor Biology Laboratory, Hospital Sant Joan de Déu, Fundación Sant Joan de Déu, Barcelona, Spain; 2 Department of Anatomic Pathology, Pharmacology and Microbiology, University of Barcelona, Barcelona, Spain; 3 Laboratory of Biostatistics and Epidemiology, Universitat Autònoma de Barcelona, Barcelona, Spain; 4 Clinical Pharmacology Service, IDIBAPS, Hospital Clinic, Barcelona, Spain; 5 Hematopathology Unit, Hospital Clinic, Barcelona, Spain; 6 Department of Pathology, Hospital Sant Joan de Déu, Barcelona, Spain; 7 Cancer Epigenetics and Biology Program (PEBC), Bellvitge Biomedical Research Institute (IDIBELL),L'Hospitalet, Barcelona, Spain; 8 Department of Physiological Sciences II, School of Medicine, University of Barcelona, Barcelona, Spain; 9 Institució Catalana de Recerca i Estudis Avançats (ICREA), Barcelona, Spain; University of Navarra, Spain

## Abstract

Neuroblastoma (NB) pathogenesis has been reported to be closely associated with numerous genetic alterations. However, underlying DNA methylation patterns have not been extensively studied in this developmental malignancy. Here, we generated microarray-based DNA methylation profiles of primary neuroblastic tumors. Stringent supervised differential methylation analyses allowed us to identify epigenetic changes characteristic for NB tumors as well as for clinical and biological subtypes of NB. We observed that gene-specific loss of DNA methylation is more prevalent than promoter hypermethylation. Remarkably, such hypomethylation affected cancer-related biological functions and genes relevant to NB pathogenesis such as *CCND1*, *SPRR3*, *BTC*, *EGF* and *FGF6*. In particular, differential methylation in *CCND1* affected mostly an evolutionary conserved functionally relevant 3′ untranslated region, suggesting that hypomethylation outside promoter regions may play a role in NB pathogenesis. Hypermethylation targeted genes involved in cell development and proliferation such as *RASSF1A*, *POU2F2* or *HOXD3*, among others. The results derived from this study provide new candidate epigenetic biomarkers associated with NB as well as insights into the molecular pathogenesis of this tumor, which involves a marked gene-specific hypomethylation.

## Introduction

Neuroblastoma (NB), the most common extracranial tumor of childhood, is a complex developmental malignancy characterized by numerous biologically significant genetic alterations that are intimately associated with the clinical outcome of patients [1]. In addition to genetic changes, the complex and heterogeneous clinical evolution of NB greatly depends on patient's age at diagnosis, as well as clinical stage and histopathologic features of the tumor [1].

Altered DNA methylation patterns have been widely reported to be a critical factor in cancer development and progression. In particular, regional DNA hypermethylation (hyperM) of CpG islands in promoter regions of tumor suppressor genes as well as global hypomethylation (hypoM) affecting DNA repeats are considered to be the most frequent cancer-related epigenetic changes [2], [3]. So far, most studies have focused their attention on the role and mechanisms of promoter hyperM, since loss of global DNA methylation was believed to affect DNA repeats and to be mainly involved in structural-nuclear functions such as chromosomal instability [2].

Although the genomic profile of NB is well-characterized, DNA methylation changes have not been extensively studied in these tumors. Several genes have been reported as being methylated in NB [4]–[9], nevertheless the genome-wide DNA methylation pattern of NB is still greatly unknown. The aim of the present study was to investigate the pattern of epigenetic changes in NB at the genome-wide level using DNA methylation-specific microarrays. Besides identifying changes globally associated with NB, we characterized DNA methylation patterns associated with distinct clinical and biological subtypes of the disease. Interestingly, we observed that gene-specific loss of DNA methylation is more prevalent than promoter hyperM. Such hypoM affected cancer-related biological functions and genes relevant to NB pathogenesis such as *CCND1* [10].

## Materials and Methods

### Patients and samples

A total of 25 primary neuroblastic tumors (NT) including 22 NBs, 2 ganglioneuromas (GN) and 1 ganglioneuroblastoma (GNB) were used for genome wide methylation analysis. Additionally, an independent cohort of 13 NBs and 2 GN was used for bisulfite pyrosequencing, mRNA gene expression and DNA copy number variation analyses (Table 1 and Table S1A). GN and GNB as well as normal human fetal brain (FB) and adrenal gland (AG) tissues were used as reference samples. NB risk assessment was defined by the International Neuroblastoma Staging System (INSS) [11]. Tumor samples were assessed by a pathologist (M.S.), only tumors with >70% viable tumor cell content were included in the study. DNA was isolated from snap-frozen samples using Cell Lysis Solution (Promega, USA) and proteinase K (Sigma, USA) following manufacturers' protocols.

**Table 1:** Patients' clinical and biological characteristics.

Characteristics	Methylation Array Samples (n = 22)	Independent Sample Set (n = 13)
Age, months		
Median	28,9	32,6
Range	0–120	0–216
INSS, n (%)		
Stage 1–3	11 (50)	5 (38,5)
Stage 4	7 (31,8)	6 (46,2)
Stage 4S	4 (18,2)	2 (15,3)
*MYCN* status, n (%)		
Amplified	5 (22,7)	10 (76,9)
Non-amplified	16 (72,8)	3 (23,1)
Undetermined	1 (4,5)	0

The NB cohort used for genome-wide methylation analysis (Methylation Array Sample Set) as well as the NB cohort used for bisulfite pyrosequencing, mRNA gene expression and DNA copy number variation analyses (Independent Sample Set) are reported in the table.

Ethics statement: The study was approved by the Institutional Research Ethics Committee (Comité Ético de Investigación Clínica, Fundación Sant Joan de Déu – CEIC-FSJD). Patients/parents/guardians signed an informed consent before collection of samples.

### Genome-wide DNA methylation profiling

DNA methylation profiling was performed using the Infinium HumanMethylation27 BeadChip (Illumina, USA). Genomic DNA bisulfite conversion and hybridization to the platform was performed at the Human Genotyping Unit at the Spanish National Cancer Center (CEGEN-CNIO, Madrid, Spain), as previously described. Data were analyzed using the BeadStudio software (version 3, Illumina Inc, USA) [12], [13]. For each CpG site we calculated the beta-value (βvalue), which is a quantitative measure of DNA methylation levels ranging from 0 for completely unmethylated to 1 for completely methylated cytosines. Possible sources of biological and technical biases that could affect our results such as gender-specific and low quality CpGs were excluded from the study [14]–[16]. Methylation microarray data have been deposited at Gene Expression Omnibus data repository (GSE39626).

### Differential DNA methylation analysis

Since there is no consensus strategy for differential methylation analysis, we used three different approaches. First, CpG sites were categorized as hyperM when βvalues were <0.25 in the reference samples and >0.75 in at least 10% of NB samples, and hypoM when βvalues were >0.75 in the reference samples and <0.25 in at least 10% of NB samples. Second, differential methylation was defined as mean βvalues between NB and reference samples showing an absolute difference greater than 0.25 [17]. Finally, an unpaired t-test was performed using Step Down Permutation (SDP) [18] and False Discovery Rate (FDR) analyses [19]. Venn diagrams were used to compare lists of differentially methylated CpGs (http://www.pangloss.com/seidel/Protocols/venn.cgi) and only those concomitantly identified by all three classification criteria were defined as differentially methylated.

### Hierarchical clustering and principal component analysis

Unsupervised and supervised agglomerative hierarchical clustering were performed using the Cluster Analysis tool from Bead Studio (version 3, Illumina Inc, USA). Principal Component Analysis (PCA) was performed with R (www.r-project.org) using the FactoMineR package available through Bioconductor.

### Bisulfite pyrosequencing

To validate DNA methylation data, bisulfite pyrosequencing (BPS) analysis was performed as previously described [20]. Briefly, genomic DNA was bisulfite converted using EpiTect Plus Bisulfite Conversion Kit (Qiagen, Hilden, Germany) according to manufacturer's instructions. A subsequent PCR amplification was performed using biotinylated primers (Table S1B). Pyrosequencing and data analysis were performed with the pyrosequencer analyzer PyroMark Q96 (Qiagen, Hilden, Germany) according to manufacturer's instructions.

### Gene expression analysis

To assess expression levels of differentially methylated genes, publicly-available expression microarray data sets [21]–[23] with representative NB tumor spectra were analyzed. Raw data was normalized to a z-score transformation. Unpaired t-test analysis adjusted by SDP and FDR was performed. Genes with a statistically significant differential expression (*p*<0.01), z-score >1 in >50% of samples, were considered differentially expressed.

For candidate genes, total RNA isolation and gene expression quantification was performed for 10 cases included in the methylation array and an independent set of 13 NB samples using quantitative real time polymerase chain reaction (qRT-PCR) as previously described [22] (Table S1B).

### Bioinformatic annotation of differentially methylated genes

The Database for Annotation, Visualization and Integrated Discovery (DAVID) v6.7 (http://david.abcc.ncifcrf.gov/) was used for gene-annotation enrichment analysis and biological pathway mapping [24]. Probability (Benjamini-Hochberg correction) lower than 0.05 was considered statistically significant.

Differentially methylated genes were classified according to their chromosomal localization. Probability distribution analysis was performed to determine potential chromosome enrichment. *P*-value<0.05 was considered statistically significant.

Promoter classification of differentially methylated genes into promoters with high (HCP), intermediate (ICP), low (LCP) and mixed CpG content, as well as the identification of Polycomb (PcG) target genes was performed as previously reported [14].

Hypergeometric probability distribution analysis with a probability cut-off <0.05, was performed to determine hyperM or hypoM chromosome enrichment and to determine promoter type and PcG-mark enrichment in differentially methylated genes. Analyses were performed with SPSS version 15.0 (SPSS, Inc, Chicago, IL).

## Results

### DNA methylation profiling and identification of differentially methylated genes in NB

In order to investigate the pattern of DNA methylation in NB, we analyzed 22 primary NB tumors using the Infinium HumanMethylation27 BeadChip microarray. Two GN, 1 GNB, as well as, normal human FB and AG tissues were used as reference samples to identify differentially methylated genes specific for NB.

We initially performed a quality control of the data obtained from the microarray analysis and excluded 3337 gender-specific and low quality CpGs. Additionally, one NB sample (NT18, Table S1A) was excluded because of poor detection *p*-values.

**Figure 1:**
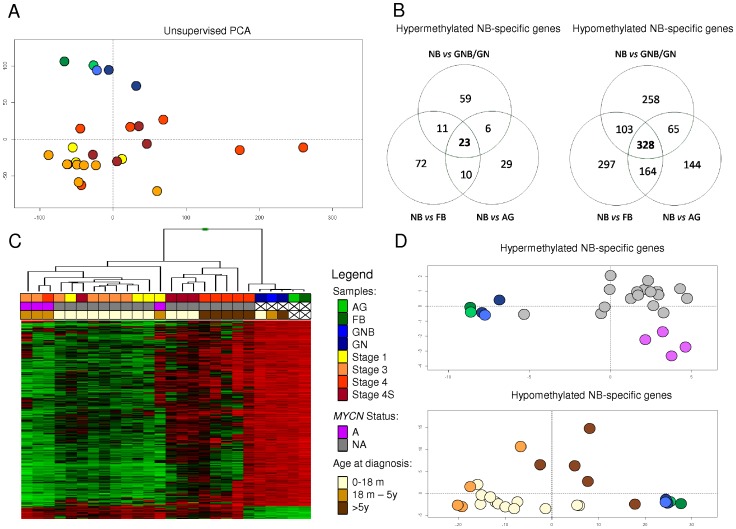
DNA methylation profiling and identification of differentially methylated genes in NB. **A**: Unsupervised Principal Component Analysis (PCA) of array-based DNA methylation data in 21 neuroblastomas (NB) (classified according to INSS Stage), 2 ganglioneuroma (GN), 1 ganglioneuroblastoma (GNB); and normal reference samples: fetal brain (FB) and adrenal gland (AG). **B**: Venn diagram showing the strategy used to identify NB-Specific genes. Hypermethylated and hypomethylated genes in NB were determined using three different supervised analyses with distinct reference samples (FB, AG and GN/GNB). **C**: Supervised hierarchical cluster analysis of DNA methylation data from NB-specific genes in 21 NB samples, 2 GN, 1 GNB; and 2 normal reference samples: FB and AG. **D**: Supervised Principal Component Analysis (PCA) in 21 NB samples, 2 GN, 1 GNB; and normal reference samples: FB and AG for NB-specific genes.

Unsupervised Principal Component Analysis (PCA) performed on all the samples included in the study, showed that NBs display a clearly distinct DNA methylation profile as compared to normal reference samples (FB and AG) and the clinically less aggressive GNB and benign GN (Figure 1A), being these two entities epigenetically undistinguishable from normal reference samples. In order to identify differentially methylated genes in NB, supervised analyses were performed using independently three different reference samples (FB, AG and 2 GN with 1 GNB). By comparing gene lists generated by these analyses we were able to identify a common set of 351 genes, being 23 hyperM and 328 hypoM, in NB (Figure 1B, Table S2A). This set of genes will be hereafter referred as NB-specific genes. We then performed a supervised hierarchical cluster and a PCA using the hyperM and hypoM gene sets separately. Remarkably, the DNA methylation pattern of hyperM genes allowed us to differentiate NBs with diverse *MYCN* amplification status. Interestingly, hypoM genes segregated NBs by their age at diagnosis, clustering those with 5 or more years separately from younger patients (Figure 1C and 1D).

The NB-specific genes were ranked according to the percentage of samples found differentially methylated and the level of βvalue changes. The most clearly hyperM genes in our series were *EMP1*, *RASSF1A*, *ACTC*, *GNG12*, *HOXD3*, *PAMR1*, *IL17RC*, *CARD11*, *POU2F2* and *P2RY6* (Table S2B). Among these, *RASSF1A* and *POU2F2* have previously been described methylated in NB tumors and cell lines. Here, both genes showed clearly increased methylation levels in ≥80% of the NB samples, which is consistent with prior reports [5], [7], [25]. Besides, to our knowledge only *HOXD3* has previously been described hyperM in cancer, specifically in prostate carcinoma [26], [27].

**Figure 2:**
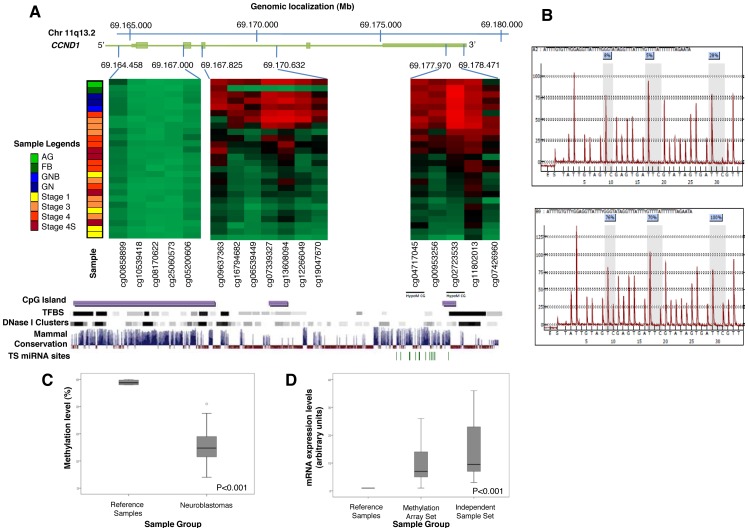
Methylation and expression data of *CCND1*. **A**: Graphical display of the DNA methylation levels of the 17 CpGs measured across the *CCND1* length. The heatmap shows the data from 21 neuroblastoma (NB) and reference samples (2 GN, 1 GNB; 1 FB and 1 AG). Below the heatmap we show some genomic features of the *CCND1* locus (UCSC Genome Browser, data from the hg19 adapted to the hg18) including Transcription Factor binding sites (TFBS), evolutionary conserved DNAseI hypersensitive domain (DNAseI cluster) and Vertebrate Multiz Aligment, PhastCons Conservation (Mammal Conservation) and miRNA target sites (TS). **B**: DNA methylation-specific pyrograms for *CCND1*. The pyrogram above corresponds to a neuroblastoma sample whereas the pyrogram below corresponds to a reference sample (FB). Grey shading shows the percentage of methylation observed for the CpGs analyzed. **C**: Box-plot for DNA methylation data of *CCND1* obtained by bisulfite pyrosequencing in an independent cohort of 13 NB and 2 GN samples and reference samples. **D**: mRNA expression levels of *CCND1* analyzed by qRT-PCR in two NB independent cohorts.

Applying the same strategy, 69 genes were clearly hypoM in NB (Table S2B). Notably, among these we identified *CCND1*. This gene has been reported to be highly expressed in a significant portion (>75%) of NB tumors and cell lines [10], [28]. The 17 CpGs analyzed across the length of the *CCND1* locus revealed a complex epigenetic pattern in NB cases and control samples (Figure 2A). In NB, we observed a reduction of DNA methylation levels at 12 of 17 CpG sites and a significant hypomethylation, using stringent criteria, only at two CpGs (Target IDs cg04717045 and cg02723533). Unexpectedly, DNA methylation loss was observed outside the 5′ region of *CCND1*, which was unmethylated in NB cases and control samples. Hypomethylation within the gene-body was not considered significant due to epigenetic heterogeneity in the reference samples (Figure 2A). The 3′ untranslated region (3′-UTR), in contrast, was consistently methylated in reference samples and hypomethylated in a large fraction of NBs. At the two significantly hypoM sites, 17 of 21 NB lose methylation as compared to all the reference samples (βvalue >0.90), being 11 of them markedly hypoM (Figure 2A).

### DNA methylation changes in clinically and biologically relevant NB subgroups

A supervised approach was used to analyze differential DNA methylation profiles between clinical and biologically relevant NB subgroups.

High-risk (HR) NBs (n = 9), defined as stage 4 and *MYCN* amplified (*MYCN* A) tumors, were compared with low-risk (LR) NBs (n = 8), which include stage 1 to 3 *MYCN* non-amplified (*MYCN* NA) tumors. A total of 19 clearly differentially methylated genes were identified. Five genes were hyperM in HR with respect to LR NB and reference samples, whereas no hypoM gene was observed in this clinical subgroup. In contrast, hyperM was not observed in LR NBs whilst 14 genes exhibited *de novo* loss of methylation in this clinically favorable subgroup (Table S2C).

We next compared the two clinically relevant metastatic NB subgroups, i.e. stage 4 (n = 6) and stage 4S (n = 4) NBs. We identified a total of 9 differentially methylated genes. Of these, 2 genes were hyperM in at least 3 of 4 stage 4S NBs and no hyperM was detected in stage 4 NBs. With regard to hypoM, 5 and 2 genes were observed in stage 4S and 4 NBs, respectively (Table S2C).

Comparing *MYCN* A (n = 5) and NA (n = 15) tumors, we identified 23 differentially methylated genes (7 hyperM and 16 hypoM) in *MYCN* amplified with respect to non-amplified tumors and reference samples (Table S2C).

Finally, we compared NBs based on patient's age at diagnosis using the clinically-established 18 month age cut-off, i.e. <18 months (n = 11) and ≥18 months (n = 10). According to our selection criteria, no consistently differentially methylated genes were identified, most likely due to the high heterogeneity in both subgroups. Based on the supervised PCA analysis shown in Figure 1D, we observed that patients with 5 or more years of age segregated separately from the younger patients, suggesting different underlying methylation patterns. A supervised analysis was thus performed using two age cut-offs, 18 months and 5 years (i.e. 0 to <18 months (n = 12), ≥18months to <5 years (n = 4), ≥5 years (n = 5)). Differences between the methylation patterns of the three age subgroups were observed. However, methylation was heterogeneous in patients younger than 5 years, but clearly distinct from the older NB patients (Table S2C). Applying only a 5 year age cut-off (<5 years, n = 16 and ≥5 years, n = 5), we identified a clear DNA methylation pattern characterized by a set of genes with a consistent loss of DNA methylation in patients younger than 5 years of age compared with the older patients, the latter being similar to reference samples (Table S2C).

### Technical and clinical validation of candidate genes by pyrosequencing

DNA bisulfite pyrosequencing of 3 NB-specific candidate genes (*EMP1*, *GNG12* and *CCND1*) as well as of *EPSTI1*, which is differentially methylated in clinically relevant NB subgroups, was performed to validate DNA methylation array data. Two NB tumors included in the microarray as well as an independent cohort of 13 NB and 2 GN samples were used for this aim. First, the degree of correlation between microarray DNA methylation and BPS data was tested and found to be significantly high (r = 0.958, *p*<0.001) (Figure S1).

Consistent with the array results, *EMP1* and *GNG12* NB-specific genes showed high methylation levels compared to reference samples in all the NB cases of the validation set (n = 13) (Figure S2B and S2C). *CCND1* showed a clear loss of methylation in 10 of the 13 independent NBs tested, which is in line with the proportion of hypoM cases identified by the methylation array (Figure 2A, 2B and 2C, Figure S2A). *EPSTI1* methylation analysis in the validation series also confirmed the array data (Figure S2D).

### Association between DNA methylation and gene expression

To investigate whether differential DNA methylation in NB is associated with gene expression, we analyzed published gene expression data of independent and representative NB tumor sets [21]–[23]. Expression data was available for 13 of 23 hyperM and 136 of 328 hypoM NB-specific genes (Table S3A). Five of thirteen (38.4%) hyperM genes showed lower expression levels as compared to reference samples. In contrast, 10 of 136 genes (7.3%) with lower methylation levels were highly expressed in NB as compared to reference samples. These results are in line with previous studies reporting that only a fraction of genes with promoter hypomethylation show a significant increase in gene expression, being, possibly, conditioned to specific activation [29]–[30].

To validate if differential DNA methylation in NB is associated with gene expression changes, we analyzed 5 candidate genes by qRT-PCR in 23 NBs with available RNA (10 cases included in the methylation array and an independent set of 13 NB samples). In general, hyperM genes showed lower expression levels in NB samples compared to reference samples. Likewise, most hypoM genes showed higher expression levels in the NB tumors with respect to reference samples, confirming microarray data (data not shown). *CCND1* was found to be highly expressed in all NB samples, consistent with previous reports [10], [31] (Figure 2D).

### Biological and functional features of differentially methylated genes in NB

Differentially methylated genes were functionally characterized using bioinformatic approaches. A gene ontology (GO) analysis of genes hypoM in NB allowed the identification of significantly enriched (*p*<0.05) functions such as defense response, immune response, immune system process, response to stimulus and epidermis development (Table S3B). Due to the small sample size, gene sets derived from other comparisons did not result in any significantly enriched GO term.

Most studies in adult tumors have reported that hyperM genes are enriched in promoters with high CpG content (HCP) and PRC2 target genes in embryonic stem cell (ESC) [17]. Remarkably, in our series, the group of hyperM genes showed a loss for HCP promoters (21.7% *vs.* 53.5% in the background, *p* = 0.072) and an enrichment for ICP promoters (34.78% *vs.* 11.68% in the background, *p* = 0.012). No significant enrichment for PRC2 targets was observed (13% *vs.* 9.6% in the background, *p* = 0.49). In contrast, hypoM genes showed the previously reported pattern [17], i.e. they were associated with an increase for low CpG content promoters (LCP) (68.3% *vs.* 22.6% in the background, *p*<0.001) and a decrease of PRC2 targets (3.3% *vs.* 9.6% in the background, *p*<0.001) (Table S3C).

To determine if differential methylation in NB occurs homogeneously throughout the genome, the chromosome distribution of NB-specific genes was analyzed. Given the small number of hyperM genes no significant enrichment or grouped tendency was observed (Table S3D). Conversely, a significant portion of hypoM genes mapped to chromosomes 1 (48 genes), 17 (25 genes), 19 (58 genes) and 21 (9 genes) (*p*<0.05 for all) and showed chromosome specific localization. These genes mapped to specific chromosome regions 1p36 (20%) and 1q21 (25%), chromosome 17p13 and 17q21 (both 27%), whereas the majority of genes identified on chromosome 19 were restricted to 19p13 (>70%; 43 of 58), and all chromosome 21 hypoM genes mapped to 21q22 (9/9).

## Dicussion

In the present study, we have analyzed the pattern of differential methylation in primary NB samples using microarray-based DNA methylation analysis. Unsupervised PCA showed that DNA methylation profiles in NB are clearly distinct from those of the clinically less aggressive GNB and benign GN and the normal reference samples used in this study (Figure 1A). Differential DNA methylation analysis using stringent criteria enabled us to identify DNA methylation changes characteristic of NB tumors. Interestingly, the pattern of hyperM and hypoM was found to be associated with clinic-biologically relevant subgroups of NB tumors. Specifically, the DNA methylation pattern of hyperM genes allowed us to differentiate NBs with diverse *MYCN* amplification status. HypoM genes segregated cases according to age at diagnosis, clustering those with 5 or more years separately from younger patients (Figure 1D). Supervised DNA methylation analysis comparing well-known NB clinical subgroups confirmed the existence of differentially methylated genes between high and low risk tumors, *MYCN* A from NA tumors as well as stage 4 from stage 4S NB. Previous reports analyzing specific candidate genes have identified hypermethylated genes associated with *MYCN* amplification status in NB cell lines and tumors [4], thus corroborating the existence of subgroup-specific DNA methylation patterns in NB. However, no consistent DNA methylation differences were identified when comparing subgroups using the clinically established prognostic age cut-off of 18 months. In contrast, in line with the analysis shown in Figure 1D, a consistent loss of DNA methylation was observed in patients younger than 5 years as compared to older patients and reference samples (Table S2C). In NB, age at time of diagnosis is a powerful marker of tumor behavior, and is thus critical in the prognostic evaluation of this developmental malignancy [32]. Traditionally, patient's age has been analyzed as a binary function, with a cut-off point initially established at 12 months and recently set at a more optimal prognostic age cut-off of 18 months [32]. However, the International Neuroblastoma Pathology Classification (the Shimada system) evaluates the prognostic impact of the histological features of the tumor considering two age cut-offs at time of diagnosis: 18 months and 5 years [33]. Interestingly, although we observed age-related DNA methylation patterns recalling these two age cut-offs, they were more consistent using the 5 years cut-off.

Overall, we observed that loss of DNA methylation is more prevalent than promoter hyperM in NB. This is in line with the well known global hypomethylation of cancer cell DNA, generally believed to affect repetitive sequences and satellite DNA and to contribute to the generation of chromosomal instability [2]. Therefore, gene-specific hypomethylation has not been extensively studied. Interestingly, we observed that hypoM affected genes relevant to NB pathogenesis such as *CCND1*. Cyclin D1 is a regulator subunit of cyclin-dependent kinases required for cell cycle G1/S transition that has been described highly expressed in various types of solid tumors as well as in more than 75% of NBs. The cause of *CCND1* overexpression in these tumors is greatly unknown. High-level amplifications of *CCND1* have been reported only in a small percentage (2%) of NB tumors [10]. Therefore, mechanisms other than gene amplification seem to be responsible for increased *CCND1* expression [34]. Recently, *GATA3*, a transcription factor overexpressed in NB, has been reported to be implicated in *CCND1* overexpression [35]. In this study, we observed loss of *CCND1* gene methylation in more than 70% of the NB tumors analyzed, associated with high *CCND1* expression levels and absence of gene amplification (data not shown). Differentially methylated CpGs were not localized in the promoter region but in the 3′ untranslated region (Figure 2A). Interestingly, based on the data available in the UCSC Genome Browser (GRCh37/hg19), the 3′-UTR of *CCND1* is an evolutionary conserved DNAseI hypersensitive domain highly enriched for microRNA target sites and Transcription Factor binding sites, including *GATA3*, *MYC*, *FOXA1/2* and *JUND*, among others. Experimental data supporting the functional role of the 3′-UTR region in the expression of *CCND1* have been reported in mantle cell lymphomas (MCL), where in addition to the fusion of *CCND1* gene on chromosome 11 to the immunoglobulin heavy chain enhancer, the loss of the 3′-UTR has been linked to hyper-proliferative MCL [36]. However, *CCND1* rearrangements leading to loss of the 3′-UTR have been observed only in a very small percentage of NBs [10]. Noteworthy, in our series *CCND1* hypoM occurs in the presence of *RASSF1A* promoter hyperM. Selective epigenetic silencing of *RASSF1A* is a common event in human cancer, including NB where it has been reported hypermethylated in >75% of tumors [5], [25]. Ras association domain family 1 isoform A is a tumor suppressor that negatively regulates cell proliferation by inhibiting cyclin D1 protein accumulation through posttranscriptional mechanisms [37]. *RASSF1A* hyperM has previously been inversely associated with cyclin D1 expression and tumor cell proliferation [38]. It is thus tempting to speculate that loss of *CCND1* gene methylation in the 3′-UTR regulatory region and concomitant hypermethylation of *RASSF1A* could represent a potential mechanism underlying *CCND1* gene overexpression in NB.

Loss of methylation also affected genes with cancer-related biological functions i.e. *SPRR3*, previously reported as hypomethylated in cancer, specifically in hepatocellular carcinoma [39]. Overexpression of *SPRR3* has been reported to promote breast and colon cancer proliferation by enhancing p53 degradation via the *AKT* and *MAPK* pathways [40], [41]. HypoM also affected genes reported to stimulate cell proliferation, i.e. *BTC*, *EGF* and *FGF6*. Betacellulin, member of the EGF family, has recently been reported to induce proliferation of neural stem cells and prevent spontaneous differentiation in cell culture via both the EGF receptor (*EGFR*) located in NSCs and *ErbB4* on neuroblasts [42]. The epidermal growth factor also acts via the EGFR to stimulate cell proliferation and neoplastic transformation. Fibroblast growth factor 6, a FGF family member, is implicated in self-renewal and maintenance of pluripotency of ES and iPS cells [43].

On the other hand, epigenetically suppressed genes included genes involved in tissue development and differentiation. Among these, *POU2F2*, previously described as being hypermethylated in NB tumors and cell lines, encodes a transcription factor involved in neuronal differentiation [7], [44]. Homeobox D3, member of the Hox gene family, has been described associated with the modulation of cell-adhesive properties during embryonic development [45]. Aberrant methylation of *HOXD3* has not been described previously in NB, but has recently been reported as a novel biomarker of prostate cancer progression together with *RASSF1A*, *TGF-β* and *APC* [27]. Differentiation-related genes included *IRF6*, which encodes a transcription factor that regulates craniofacial development and epidermal proliferation. *IRF6* downregulation has been correlated with promoter methylation in invasive squamous cell carcinomas [46]. Epithelial membrane protein-1, a member of the peripheral myelin protein 22 family expressed in first differentiating neurons, has been described involved in neural differentiation [47]. Downregulation of *EMP1* has been described in squamous cell lung carcinoma suggesting a potential tumor suppressor function [48].

The functional characterization of differentially methylated genes revealed that hyperM occurs predominantly at ICP promoters, while hypoM affects mostly LCP genes, regardless of the NB subgroup studied. Moreover, the vast majority of changes of DNA methylation were observed in genes that are not targets of PcG proteins in ESCs. These findings are in contrast with most other tumors in which *de novo* methylation affects predominantly genes with dense CpG island promoters highly enriched for PcG targets [14], [16], [17]. The epigenetic features of NB identified in this study may be related to the embryonic origin of this developmental disease, fundamentally different from that of adult cancers. Additionally, chromosome distribution of NB-specific genes showed that global DNA hypoM in NB is organized not only at the functional level but also at spatial level since it significantly affects specific chromosomes as well as chromosomal regions previously described to be recurrently altered in NB.

This study provides a genome-wide view of the DNA methylation landscape in NB. However, it is worth mentioning that our conclusions are based on a small cohort of cases which may have led to an overestimation of the data, and that the array used does not differentiate 5-methylcytosine from 5-hydroxymethylcytosine. In spite of these caveats, our findings suggest that hypoM is a prevalent epigenetic alteration in NB that affects cancer-related biological functions and specific genes relevant for NB pathogenesis, such as *CCND1*. Moreover, hypomethylation involves chromosomal regions recurrently altered in this malignancy. Our findings also provide evidence for the existence of DNA methylation profiles characteristic of NB. We identified specific DNA methylation changes associated with clinical and genetic features of NB tumors, suggesting altered DNA methylation patterns as a critical factor for NB tumorigenesis.

## Supporting Information

Figure S1 **Technical validation of methylation analysis.**
(TIFF)

Figure S2 **Biological validation of methylation analysis.**
(TIFF)

Table S1 Table S1A: Clinical and biological data of samples. Table S1B: Primer information.
(XLSX)

Table S2 Table S2A: Methylation status of the CpG dinucleotide analyzed to identify NB-specific genes. Table S2B: NB-specific ranked genes. Table S2C: Differentially methylated genes in NB subgroups.
(XLSX)

Table S3 Table S3A: Gene expression data from microarrays of NB-specific genes. Table S3B: Gene-ontology terms enrichment for differentially methylated genes. Table S3C: Polycomb Repressive Complex 2 (PCR2) target genes and promoter class distribution of NB-specific genes. Table S3D: Chromosomal distribution of NB-specific genes.
(XLSX)
