# Supplementary figures and images for: DNA Hypomethylation Affects Cancer-Related Biological Functions and Genes Relevant in Neuroblastoma Pathogenesis
Source: PLoS One. 2012 Nov 7;7(11):e48401. doi: 10.1371/journal.pone.0048401 (PMC3492354; doi:10.1371/journal.pone.0048401)

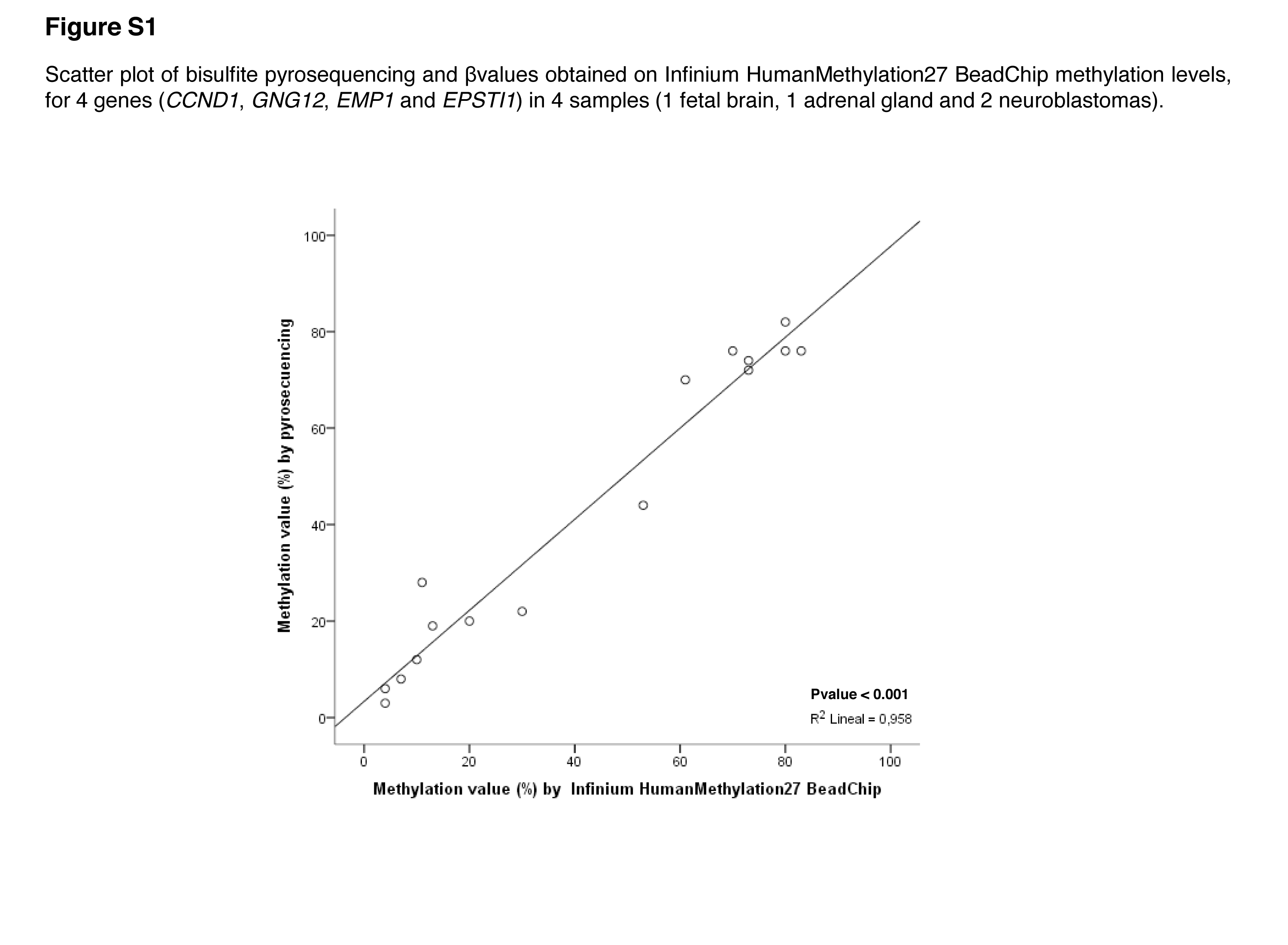

Supplement: Figure S1 — Technical validation of methylation analysis. (TIFF) [file pone.0048401.s001.tiff]

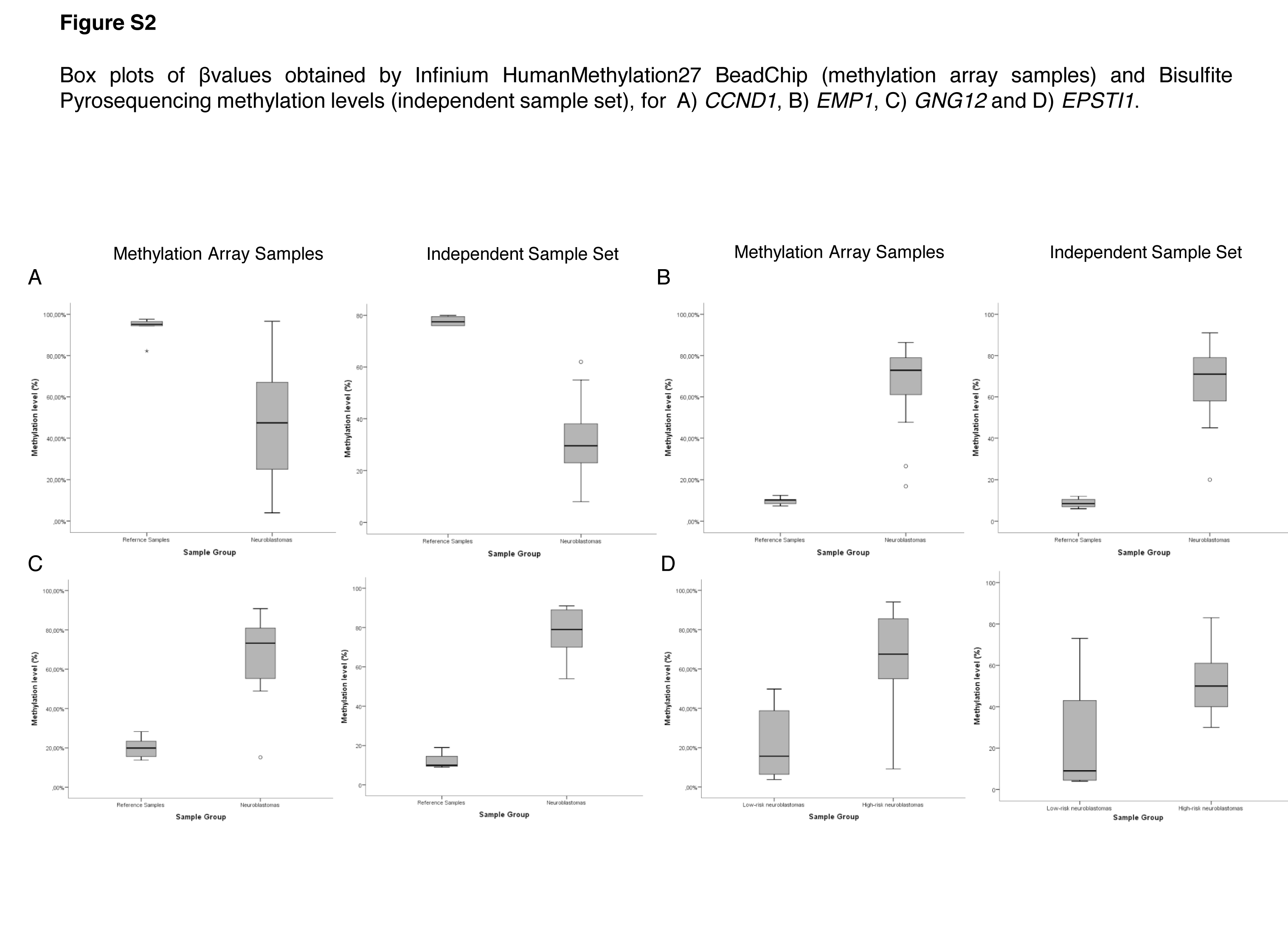

Supplement: Figure S2 — Biological validation of methylation analysis. (TIFF) [file pone.0048401.s002.tiff]
